# Docetaxel versus androgen receptor pathway inhibitors as first-line therapy for metastatic castration-resistant prostate cancer after doublet therapy: a multicenter retrospective study from Saudi Arabia

**DOI:** 10.1186/s12894-025-02019-8

**Published:** 2025-12-22

**Authors:** Shouki Bazarbashi, Ahmed S. Abdelmotal, Tarek Arabi, Waleed Fallatah, Noura Alzannan, Tusneem Elhassan, Mohamed Aseafan, Mohammed Mahroos, Maram Aljishi, Albatool Busbaih, Rajaa Aldandan, Muhammad Shahzad Rauf, Fahad A Almugbel, Faisal Azam, Mubarak Almansour

**Affiliations:** 1 Department of Medical Oncology, Cancer Center of Excellence, King Faisal Specialist Hospital and Research Center, Riyadh, Saudi Arabia; 2 Faculty of Medicine, Ain Shams University, Cairo, Egypt; 3 College of Medicine, Alfaisal University, Riyadh, Saudi Arabia; 4 Section of Medical Oncology, Department of Internal Medicine, Security Forces Hospital, Riyadh, Saudi Arabia; 5 Department of Adult Medical Oncology, Princess Noorah Oncology Centre, Ministry of National Guard Health Affairs-Western Region (MNGHA-WR), King Abdullah International Medical Research Centre (KAIMRC), King Saud Bin Abdulaziz University for Health Sciences (KSAU-HS), King Abdulaziz Medical City, Jeddah, 21423 Saudi Arabia; 6 Department of Medical Oncology, King Fahad Specialist Hospital, Eastern Health Cluster, Dammam, Saudi Arabia

**Keywords:** Metastatic castration-resistant prostate cancer, ARPI, Docetaxel, Treatment sequencing, Real-world data

## Abstract

**Background:**

The management of metastatic castration-resistant prostate cancer (mCRPC) following progression on androgen deprivation therapy (ADT) combined with androgen receptor pathway inhibitors (ARPI) in the castration-sensitive (CS) setting remains unclear. Limited data exist comparing the efficacy of ARPI versus docetaxel as first-line treatment in this context.

**Methods:**

We conducted a retrospective multicentre study across three tertiary cancer centres in Saudi Arabia, including 60 patients with pathologically confirmed mCRPC who progressed after doublet therapy (ADT + ARPI) in the CS setting between January 2018 and September 2022. Patients received either ARPI (abiraterone acetate or enzalutamide) or docetaxel as first-line therapy for mCRPC. Primary endpoints were prostate-specific antigen (PSA) response rate and biochemical progression-free survival (PFS). Secondary endpoints included overall survival (OS) and treatment patterns. PSA response was defined as ≥ 30% decline at 12 weeks from baseline. Survival analyses were performed using Kaplan-Meier and log-rank tests.

**Results:**

Among 60 eligible patients (median age 65 years), 28 received ARPI and 32 received docetaxel. PSA response rates were 39.3% for ARPI and 37.5% for docetaxel. Median PFS was 2.9 months (95% CI 0.37–5.5) for ARPI and 4.5 months (95% CI 2.6–6.3) for docetaxel (*p* = 0.137). Median OS was 13 months (95% CI 4.0–23.9) for ARPI and 16 months (95% CI 6.1–25.9) for docetaxel (*p* = 0.236). In patients with visceral metastases, docetaxel conferred significantly longer OS compared to ARPI (14.4 vs. 5.9 months, *p* = 0.025). Multivariate analysis identified nadir PSA in the CS setting as a predictor of PFS and first-line therapy in the CR setting, visceral metastasis and duration of therapy in CS setting as predictors of OS.

**Conclusions:**

In patients progressing to mCRPC after doublet therapy in the CS setting, docetaxel demonstrated a trend toward longer PFS and OS compared to ARPI, with a significant survival advantage in those with visceral metastases. These findings highlight the need for prospective randomized trials and biomarker-driven treatment strategies to optimize therapy sequencing in this evolving treatment landscape.

## Introduction

The management of metastatic prostate cancer has undergone significant change in the past decade. Earlier, androgen deprivation therapy (ADT) alone or in combination with first generation anti-androgens was the standard of care and gave an average median survival of 2.5 to 3 years [1]. Initial development occurred with the introduction of docetaxel in metastatic castration resistant prostate cancer (mCRPC) which resulted in survival improvement over mitoxantrone and prednisone [2]. Subsequently the COUGAR 302 and the PREVAIL trials showed that abiraterone acetate with prednisone, and enzalutamide improved survival over prednisone alone *±* placebo [3, 4] and established abiraterone acetate with prednisone or enzalutamide as the first line therapy of mCRPC. However, since androgen receptors pathway inhibitors (ARPI) including abiraterone acetate and enzalutamide were introduced in the metastatic castration sensitive (CS) setting, the efficacy of abiraterone acetate, enzalutamide and docetaxel became unclear with some data indicating reduced efficacy compared to earlier published studies [5–11]. Limited data exists on the comparative efficacy of abiraterone acetate, enzalutamide and docetaxel as first line treatment of mCRPC following doublets therapy ( ADT + ARPI) in the CS setting.

The following study evaluated the efficacy of ARPI (represented here as abiraterone acetate or enzalutamide) versus docetaxel in first line treatment of mCRPC patients following doublets therapy in the CS setting.

## Methods

This is a retrospective multicenter study from 3 tertiary care cancer centers in Saudi Arabia. The primary objective was to assess prostate specific antigen (PSA) response rate and biochemical progression free survival in patients receiving ARPI versus docetaxel in first-line setting of patients with mCRPC progressing on doublet therapy (ARPI and ADT) in the CS setting. Secondary endpoints were overall survival and treatment pattern of eligible patients. Eligible patients were 18 years and above with pathologically confirmed mCRPC who received doublet therapy (abiraterone acetate or enzalutamide) in the CS setting between January 2018 and September 2022. Patients should have received upon progression to mCRPC either abiraterone acetate, enzalutamide or docetaxel in addition to ADT. Patients who received prior docetaxel and non-metastatic castration resistant patients were excluded. Prior docetaxel doublets in CS setting were excluded since doublets ADT and ARPI were the standard of care in our practice, and the study was aimed to assess first-line efficacy of docetaxel or ARPI in castration resistant setting. Patients who received other approved ARPI in CS setting such as apalutamide were excluded as it was not registered in our centers. Electronic medical records of eligible patients were reviewed. Demographics, ARPI agent received, duration of therapy and nadir PSA level in CS setting were collected. Data in the castration resistant (CR) state including PSA level, organs involved, performance status, treatment received (docetaxel vs. ARPI) were collected.

PSA response was defined as ≥ 30% reduction in the PSA baseline value at 12 weeks. PSA progression (PD) was defined as ≥ 25% increase (and at least absolute increase by 2 ug) in the PSA value compared to nadir in responders and compared to baseline in non-responders. Patients who do not fall into the above categories are considered to have stable disease (SD). Radiographic assessment of response was not used in this analysis.

Categorical variables were summarized as frequencies and percentages, and continuous variables as the median with interquartile range (IQR). Survival probabilities were estimated by the Kaplan–Meier method and survival curves compared with the log-rank test.

Univariate Cox proportional-hazards models were fit for all candidate covariates; those with *p* < 0.20 were entered into a multivariable, stepwise Cox regression (entry *p* < 0.05, removal *p* >0.10), with treatment type forced into the model as a fixed effect. The proportional-hazards assumption was evaluated both graphically and formally: [1] scaled Schoenfeld residuals were tested for time-dependence via the Grambsch–Therneau global test (cox.zph in R), and [2] log(− log[survival]) versus log(time) plots were inspected for parallelism. Any covariate violating the assumption was subsequently included as a time-dependent term in the final model. All tests were two-sided, and statistical significance was defined as *p* < 0.05. All statistical analyses were performed using R (version 4.2.0; R Foundation for Statistical Computing, Vienna, Austria).

This research project was conducted in accordance with the ethical principles contained in the updated declaration of Helsinki (2013), Good Clinical Practice Guidelines and the policies and guidelines of the respective ethical committee of each participating hospital. Enrolled subjects remained anonymous with no identifying data or protected health information recorded. Locked cabinet in the principal investigators office was used to store and safeguard the confidentiality of the collected data. All data abstracted was that already existed in the subjects’ medical records and obtained through routine clinical practice. Subjects were not contacted for follow-up information. Waiver of consent was obtained in view of the retrospective nature of the study and Ethical Committee approval was obtained in each participating center.

## Results

A total of 60 patients were eligible for analysis. The median age was 65 years (Range 48–91). Doublets in the CS setting consisted of ADT with abiraterone acetate and prednisone in 47 patients (77%) and enzalutamide in 13 patients (23%). Upon progression to mCRPC 28 patients (46%) received ARPI (abiraterone acetate in 8 and enzalutamide in 20). The remainder (32 patients, 54%) received docetaxel. Characteristics of both groups are listed in Table 1.

**Table 1:** Characteristics of patients in docetaxel and ARPI in CR setting

Item (*n*, IQR)	Docetaxel *n* = 32	Hormonal *n* = 28	*P*-value
Age median (Range)	63 (59–70)	69.5 (62–77)	0.16
Prior local therapy (%)	8 (25)	12 (43)	0.17
Performance status (ECOG scale)			
0–1 (%)	25 (78)	16 (57)	0.17
≥ 2	7 (21.8)	12 (43)	
Disease volume at diagnosis of CSPC (%)			0.2
High	22 (68.7)	15 (53.6)	
Low	10 (31.23)	13 (46.4)	
Disease risk at diagnosis of CSPC (%)			0.07
High	22 (68.7)	13 (46.4)	
Low	10 (31.3)	15 (53.6)	
PSA at diagnosis of CSPC, median (Range)	62.5 (5.8–189)	97.5 (23.3-358.7)	0.7
Fist-line AA (%)	27 (84)	20 (71)	0.37
Median duration first-line, months (Range)	13 (7-19.2)	12,8 (5,3–23)	0.68
Visceral metastasis (%)	9 (28)	9 (32)	0.78
Baseline PSA at second line, median ng/ml (Range)	47 (9.2–134)	28 (7.6–81)	0.43
Nadir PSA at first line, median ng/ml (Range)	32 (4–88)	27 (4–86)	0.79

*ARPI* Androgen receptor pathway inhibitors, *IQR* Interquartile range, *AA* Abiraterone acetate, *PSA* Prostate specific antigen, *CR* Castration resistant, *CSPC* Castration sensitive prostate cancer, *ECOG* Eastern Oncology Cooperative Group

**Fig. 1:**
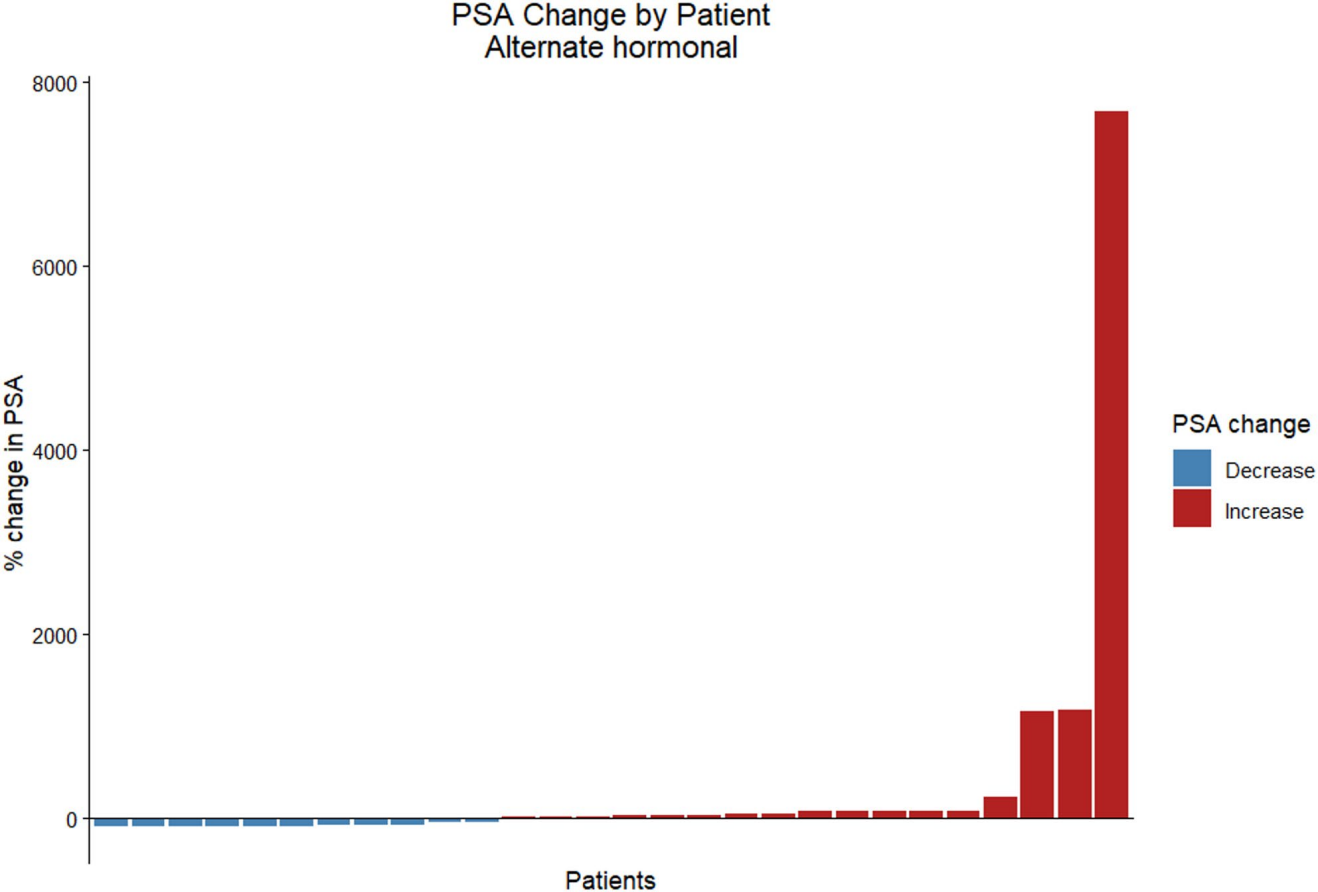
Best percentage change in baseline PSA values by patient in the alternate hormone group

**Fig. 2:**
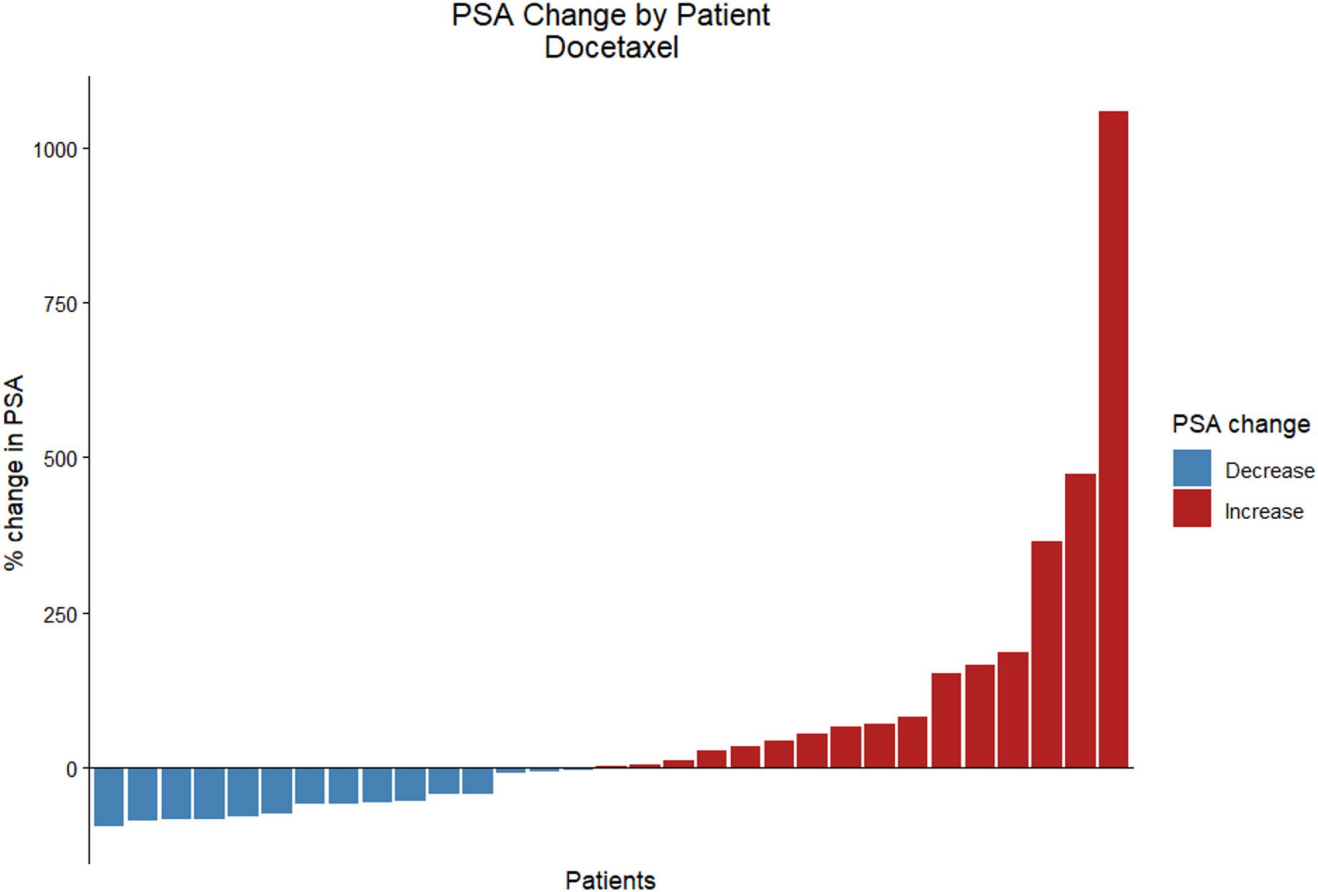
Best percentage change in baseline PSA values by patient in the docetaxel group

PSA response assessment at 12 weeks in the docetaxel group (32 patients) showed 12 responders (37.5%), 6 (18.8%) SD and 14 patients 43.8% with PD. Responses in the ARPI group were 11(39.3%), 4 (14.3%) and 13(46.4%) respectively (Figs. 1 and 2). The median follow-up on first-line CR setting was 53.5 months (95% CI 14.4–92.5) for the ARPI group and 50.7 months (95% CI 41.4–60) for the docetaxel group.

**Fig. 3:**
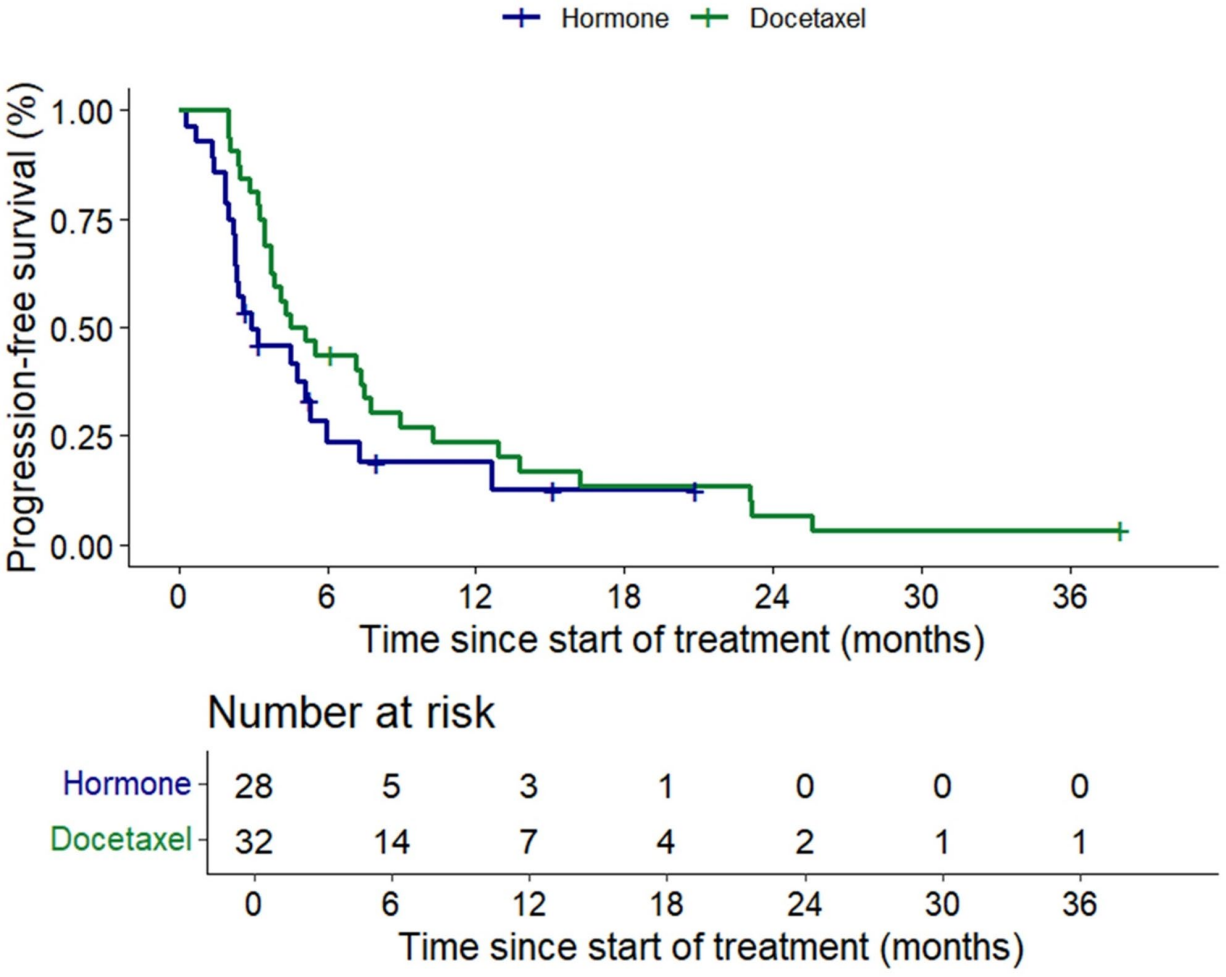
Kaplan-Meier curve of PSA progression-free survival in alternate hormone group (green line) and docetaxel group (blue line)

**Fig. 4:**
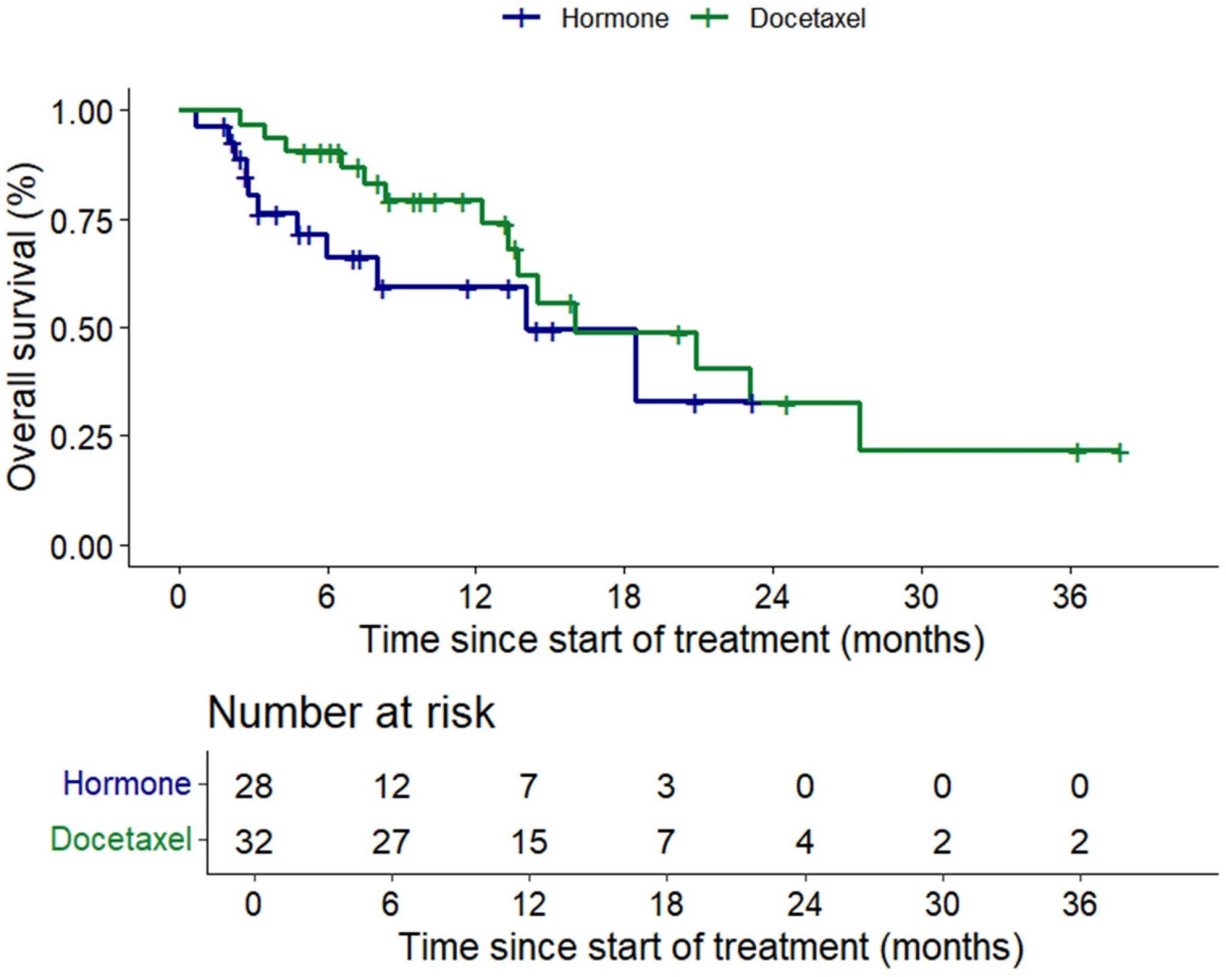
Kaplan-Meier curve of overall survival in alternate hormone group (green line) and docetaxel group (blue line)

The median PFS for the ARPI and docetaxel groups were 2.9 months (95% CI 0.37–5.5) and 4.5 months (95% CI 2.6–6.3) respectively (*p* = 0.137) (Figs. 3). Median OS similarly were 13 months (95% CI 4.0-23.9) and 16.0 (95% CI 6.1–25.9) respectively (*p* = 0.236) (Fig. 4). Median OS from start of ARPI in CS setting was 52.9 months (95% CI 6.6-99.25) for the ARPI group vs. 40.9 months (95% CI 22.26–59.61) (*p* = 0.859) for the docetaxel group.

**Fig. 5:**
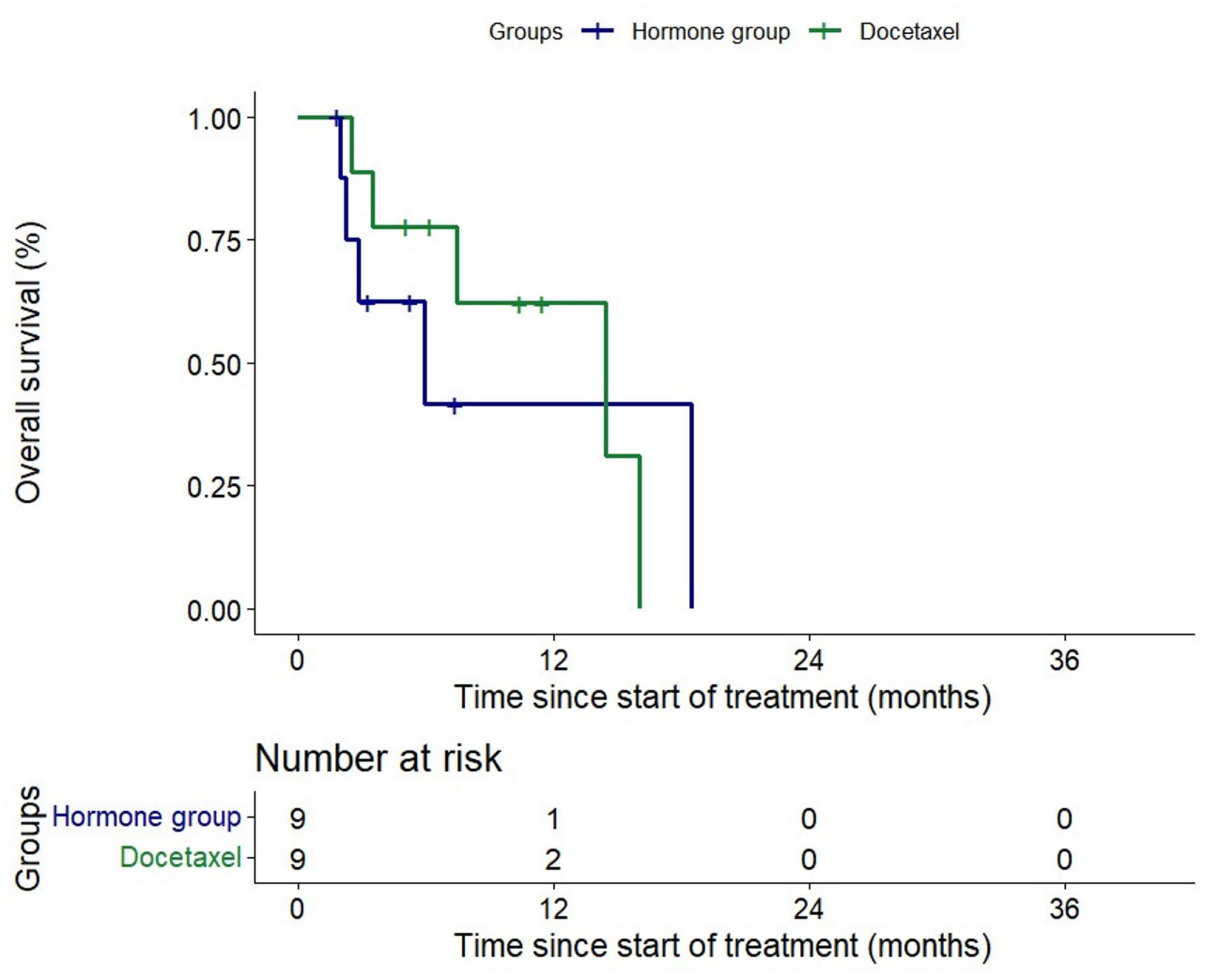
Kaplan-Meier curve of overall survival in alternate hormone group (green line) and docetaxel group (blue line) in patients with visceral metastasis

A total of nine patients were identified with visceral metastasis in each group. Median OS for the subgroup with visceral metastasis was 5.9 (95% CI 0.0-11.6) and 14.4 months (95% CI 4.0-24.9) (*p* = 0.025) for the ARPI and docetaxel groups respectively (Fig. 5).

Univariate analysis showed that the duration of therapy (< vs. > 12 months) and nadir PSA (< vs. > 30 ng/ml) in the CS setting were statistically significant for PFS while duration of therapy in CS setting and visceral metastasis were significant for OS (Tables 2 and 3). Patients without visceral metastasis showed OS of 23 months (95% CI: 10.2, 35.8) compared to 14.4 months (95% CI: 3.9–24.9) in patients with visceral metastasis (*P* = 0.01).

On the multivariate level, only nadir PSA (< vs. > 30 ng/ml) in castration sensitive setting was statistically significant for PFS (*p* = 0.002) while the type of first-line therapy in castration resistant setting (ARPI vs. Docetaxel), duration of therapy in CS setting (≤ vs. ≥ 12 months) and the presence of visceral metastasis were all significant factors for OS (*p* = 0.043, 0.017 and 0.03 respectively).

## Discussion

Data on the efficacy of alternate hormonal therapy or docetaxel in patients who fails doublets hormonal therapy in the CS setting is scarce. Hence there is no standard of care accepted in this setting and generally most guidelines do not recommend ARPI post ARPI. Most studies addressing the efficacy of ARPI post ARPI were not exclusive for ARPI in first line mCRPC. For example, in PSMAfore trial, 81% of patients enrolled had prior ARPI in the Castrate resistant setting [12]. Additionally, the CARD trial included patients who progressed within 12 months on prior ARPI before or after docetaxel [13].

The exception to the above would be in patients with homologous recombinant repair deficiency (HRRD) in whom Poly (ADP-Ribose) Polymerase (PARP) inhibitors have shown improved efficacy compared to physicians’ choice (either alternate hormone therapy or docetaxel). The TRITON-3 trial randomized patients with metastatic castrate resistant prostate cancer failing doublets hormonal therapy in the CS setting with BRCA 1, BRCA 2 or ATM alterations to rucaparib vs. physicians’ choice. The primary endpoint was radiographic PFS. At a median follow-up of 62 months, median radiographic PFS was 10.2 months in the rucaparib group versus 6.4 months for the physician’s choice group with a hazard ratio (HR) of 0.61 and a 95% confidence interval (CI) of 0.47 to 0.80; *P* < 0.001 [14]. Eighteen to 19% of the patients in this trial were on their first-line therapy for castration resistant disease, and 56% of the physicians’ choice received docetaxel. Subgroup analysis of the BRCA patients of the physician’s choice group showed median radiographic PFS of 8.3 months (6.1–9.9) for patients receiving docetaxel versus 4.5 months (3.3–5.8) for the alternate hormone group. This was used by investigators as evidence of superiority of docetaxel to alternate hormonal therapy in the castration resistant setting. However, knowing that more than 80% of the patients received ≥ 1 line of therapy in the CR setting and around 25% received prior docetaxel in the CS setting, in a specific patients’ population with BRCA or ATM alteration makes generalization of these results to unselected patients in the first-line CR setting unacceptable.

Unfortunately, most of the reported studies evaluating the efficacy of docetaxel or alternate hormonal therapy in the CR setting were not in first-line following ARPI and ADT doublets in CS setting [5–11]. Similarly, the CARD trial evaluated the efficacy of alternate hormone therapy versus cabazitaxel in patients with CRPC. The primary endpoint was radiographic PFS. Eligible patients had CRPC treated previously with docetaxel and had progressed during 12 months of treatment on an ARPI (abiraterone or enzalutamide, before or after docetaxel) most of which were in the CR setting. With a median follow-up of 9.2 months. the median radiographic PFS was 8.0 months in the cabazitaxel group and 3.7 months in the ARPI group, (HR, 0.54; 95% confidence interval [CI], 0.40 to 0.73; *P* < 0.001). The median overall survival was 13.6 months in the cabazitaxel and 11.0 months in the ARPI group (HR, 0.64; 95% CI, 0.46 to 0.89; *P* = 0.008) [13]. The trial was criticised for enrolling only patients who have progressed within 12 months of therapy on prior ARPI, hence selecting patients likely to failed hormonal therapy. Zhang et al. performed a network meta-analysis evaluating first-line therapeutic strategies in metastatic CRPC. Eligibility for inclusion was no prior docetaxel or ARPI. A total of seven randomized controlled trials (6,641 patients) were included and the meta-analysis showed significantly improved OS for both docetaxel + prednisone and cabazitaxel + prednisone compared to abiraterone acetate [15].

Wenzel et al. retrospectively evaluated patients who progressed to first-line metastatic CRPC after initial treatment for metastatic CSPC with either ARPI or docetaxel registered on the prospective FRAMCAP (Frankfurt Metastatic Cancer database of the Prostate) database. A total of 143 patients progressed to mCRPC and received first-line mCRPC therapy, of which 35% (*n* = 50) received alternate hormonal therapy after initial ARPI in the CS setting and 27% (*n* = 38) received docetaxel after initial ARPI in the CS setting. Median PFS on first-line metastatic CRPC was 10.3 months for patients receiving alternate hormonal therapy vs. 7.1 months for patients receiving docetaxel (*p* = 0.3). Also, no OS differences were observed (*p* = 0.7) [16]. PFS2 (progression-free survival after second-line therapy) was longer in the alternate hormonal therapy group after multivariable adjustment, but this did not translate into an OS advantage.

The ProBio trial compared ARPIs vs. taxanes (docetaxel/cabazitaxel) in 193 metastatic CRPC patients with detectable circulating tumor DNA. The primary endpoint was time to no longer clinically benefitting (NLCB). 18% of the study population received doublets therapy with ARPI in the CS setting. The rest received either docetaxel or had prior treatment for CR setting. While the study did not provide subgroup-specific results for the 18% who received prior ARPIs in the castration-sensitive setting, the trial reported median NLCB of 11.1 months for ARPI vs. 7.4 months for taxanes and median OS of 38.7 versus 21.7 months respectively in an unselected group of patients [17].

The largest study to date examining the efficacy of alternate hormonal therapy versus docetaxel in first-line therapy of metastatic CRPC was the retrospective real-world patient’s data extracted from Flatiron electronic health record database reported by Swami et al. This study confirmed that alternate hormonal therapy as first-line treatment for mCRPC was the most common sequence rather than docetaxel. Efficacy assessment showed in patients previously treated with hormonal doublets in CS setting, switching to another ARPI as first-line therapy for metastatic CRPC was associated with improved median OS compared to switching to docetaxel. In patients who received abiraterone in CS setting, median OS was 15.6 months with subsequent enzalutamide versus 8.7 months with docetaxel (adjusted HR for death with docetaxel vs. alternate hormonal therapy: 1.32; *p* = 0.009). Additionally in patients who received enzalutamide in CS setting, median OS was 13.2 months with subsequent Abiraterone acetate in CR setting versus 9.7 months with docetaxel (HR: 1.40; *p* = 0.009). This study supported an ARPI-ARPI sequence rather than ARPI-docetaxel upon progression to CR setting [18].

Our study hence adds to the limited available data for the optimal therapy in patients progressing on doublets ADT and ARPI used in the CS setting. Despite higher response rate with ARPI, docetaxel had numerically longer PFS and OS. This difference did not translate into statistical significance for PFS but was statistically significant for OS in multivariate level (*p* = 0.043). Difference in survival was more pronounced in patients with visceral metastasis (median OS for ARPI vs. docetaxel 5.9 vs. 14.4 months respectively) suggesting a better efficacy for docetaxel in this group of patients. This improved result in patients with visceral metastasis reflects the heterogeneity of prostate cancer tumors and the aggressiveness of cancer cells in patients with visceral metastasis making them more likely to respond to chemotherapy with likely acquired resistance to hormonal therapy.

Our study has several limitations, including its retrospective design with its inherent selection bias, the small number of patients despite being multicentre and the use of PSA value on assessing response and progression rather than radiological evaluation.

In conclusion, our study showed similar response rate and PFS with possible improved survival in patients receiving docetaxel compared to ARPI in the CR setting and unselected population. The result should be interpreted with caution. Randomized studies and biomarkers directed therapy selection should be undertaken to firmly address the best treatment and establish a standard of care.

## Data Availability

The dataset supporting the conclusions of this article is available in Dr. Shouki Bazarbashi repository and will be provided upon request through e-mail: [bazarbashi@gmail.com](mailto: bazarbashi@gmail.com) .
